# Sensitivity to Experiencing Alcohol Hangovers: Reconsideration of the 0.11% Blood Alcohol Concentration (BAC) Threshold for Having a Hangover

**DOI:** 10.3390/jcm9010179

**Published:** 2020-01-09

**Authors:** Joris C. Verster, L. Darren Kruisselbrink, Karin A. Slot, Aikaterini Anogeianaki, Sally Adams, Chris Alford, Lizanne Arnoldy, Elisabeth Ayre, Stephanie Balikji, Sarah Benson, Gillian Bruce, Lydia E. Devenney, Michael R. Frone, Craig Gunn, Thomas Heffernan, Kai O. Hensel, Anna Hogewoning, Sean J. Johnson, Albertine E. van Lawick van Pabst, Aurora J.A.E. van de Loo, Marlou Mackus, Agnese Merlo, René J.L. Murphy, Lauren Owen, Emily O.C. Palmer, Charmaine J.I. van Rossum, Andrew Scholey, Chantal Terpstra, Vatsalya Vatsalya, Sterre A. Vermeulen, Michelle van Wijk, Ann-Kathrin Stock

**Affiliations:** 1 Division of Pharmacology, Utrecht Institute for Pharmaceutical Sciences (UIPS), Utrecht University, 3584CG Utrecht, The Netherlands; j.c.verster@uu.nl (J.C.V.); k.a.slot@uu.nl (K.A.S.); kanogeianaki@gmail.com (A.A.); l.arnoldy@uu.nl (L.A.); stephaniebalikji@hotmail.com (S.B.); annahogewoning@gmail.com (A.H.); albertinevanlawick@live.nl (A.E.v.L.v.P.); marloumackus@gmail.com (M.M.); s.a.vermeulen@students.uu.nl (S.A.V.); m.wijk@students.uu.nl (M.v.W.); 2 Institute for Risk Assessment Sciences (IRAS), Utrecht University, 3584CM Utrecht, The Netherlands; 3 Centre for Human Psychopharmacology, Swinburne University, Melbourne, VIC 3122, Australia; eayre@swin.edu.au (E.A.); sarahmichellebenson@gmail.com (S.B.); andrew@scholeylab.com (A.S.); chantalterpstra92@gmail.com (C.T.); 4 Centre of Lifestyle Studies, School of Kinesiology, Acadia University, Wolfville, NS B4P 2R6, Canada; darren.kruisselbrink@acadiau.ca (L.D.K.);; 5 Addiction and Mental Health Group, Department of Psychology, University of Bath, Bath BA2 7AY, UK; sa221@bath.ac.uk (S.A.); cag35@bath.ac.uk (C.G.); 6 Psychological Sciences Research Group, University of the West of England, Bristol BS16 1QY, UK; chris.alford@uwe.ac.uk (C.A.); JohnsonS11@cardiff.ac.uk (S.J.J.); 7 Education and Social Sciences, University of the West of Scotland, Paisley PA1 2BE, UK; gillian.bruce@uws.ac.uk (G.B.); agnese.merlo@gmail.com (A.M.); 8 School of Psychology, Life and Health Sciences, Ulster University, Coleraine, Co. Londonderry BT52 1SA, UK; lydiadevenney@gmail.com; 9 Department of Psychology, University at Buffalo, The State University of New York, Buffalo, NY 14203, USA; mrf@buffalo.edu; 10 Department of Psychology, Faculty of Health and Life Sciences, Northumbria University, Newcastle upon Tyne NE1 8ST, UK; tom.heffernan@northumbria.ac.uk; 11 Cambridge Biomedical Campus, Department of Paediatrics, Addenbrooke’s Hospital, Cambridge University Hospitals NHS Foundation Trust, Cambridge CB2 0QQ, UK; kai.hensel@gmail.com; 12 Faculty of Health, Department of Paediatrics, Center for Clinical & Translational Research (CCTR), Witten/Herdecke University, 58455 Witten, Germany; 13 Centre for Trials Research, Cardiff University, Cardiff CF14 4YS, UK; 14 Department of Psychology, School of Health and Society, University of Salford, Salford 5 M6 6PU, UK; L.J.Owen2@salford.ac.uk; 15 Department of Medicine, Imperial College London, London W12 0NN, UK; e.palmer@imperial.ac.uk; 16 Department of Medicine, University of Louisville, Louisville, KY 40202, USA; vatsalya.vatsalya@louisville.edu; 17 Alcohol Research Center, University of Louisville, Louisville, KY 40202, USA; 18 Hepatobiology & Toxicology Center, University of Louisville, Louisville, KY 40202, USA; 19 National Institute on Alcohol Abuse and Alcoholism, NIH, Bethesda, MD 20892, USA; 20 Robley Rex Louisville VAMC, Louisville, KY 40206, USA; 21 Cognitive Neurophysiology Department of Child and Adolescent Psychiatry, Faculty of Medicine of the TU Dresden, University of Dresden, D-01307 Dresden, Germany

**Keywords:** alcohol, hangover, sensitivity, subjective intoxication, blood alcohol concentration

## Abstract

The 2010 Alcohol Hangover Research Group consensus paper defined a cutoff blood alcohol concentration (BAC) of 0.11% as a toxicological threshold indicating that sufficient alcohol had been consumed to develop a hangover. The cutoff was based on previous research and applied mostly in studies comprising student samples. Previously, we showed that sensitivity to hangovers depends on (estimated) BAC during acute intoxication, with a greater percentage of drinkers reporting hangovers at higher BAC levels. However, a substantial number of participants also reported hangovers at comparatively lower BAC levels. This calls the suitability of the 0.11% threshold into question. Recent research has shown that subjective intoxication, i.e., the level of severity of reported drunkenness, and not BAC, is the most important determinant of hangover severity. Non-student samples often have a much lower alcohol intake compared to student samples, and overall BACs often remain below 0.11%. Despite these lower BACs, many non-student participants report having a hangover, especially when their subjective intoxication levels are high. This may be the case when alcohol consumption on the drinking occasion that results in a hangover significantly exceeds their “normal” drinking level, irrespective of whether they meet the 0.11% threshold in any of these conditions. Whereas consumers may have relative tolerance to the adverse effects at their “regular” drinking level, considerably higher alcohol intake—irrespective of the absolute amount—may consequentially result in a next-day hangover. Taken together, these findings suggest that the 0.11% threshold value as a criterion for having a hangover should be abandoned.

Alcohol hangover is defined as the combination of mental and physical symptoms experienced the day after a single episode of heavy drinking, starting when blood alcohol concentration (BAC) approaches zero [1]. The hangover state can comprise a variety of symptoms which differ in presence and severity among drinkers [2,3]. These symptoms include, but are not limited to, nausea, sleepiness, concentration problems, and headache. In the 2010 consensus paper of the Alcohol Hangover Research Group [4], it was stated that in order to experience a hangover per se, a minimum BAC of 0.11% should be reached. In the current consensus paper, we discuss why the 0.11% threshold value as a criterion for having a hangover should be abandoned. 

BAC varies depending on the combination of the amount of alcohol consumed and drinking duration. A smaller impact is also evident for other factors such as sex and body weight. For example, a BAC of 0.11% roughly equates to consuming about 6 US standard drinks (14 g of alcohol each) or 8.4 European standard drinks (10 g of alcohol each) over a period of 2 hours [5]. This threshold was based on a study by Chapman et al. [6] in which participants experienced hangovers at this BAC level. At first glance, observing drinking levels of student samples and corresponding average BACs [3,7,8,9], the threshold seems well selected. However, a closer look at the data revealed that this threshold could well be an arbitrary one. Research on large Dutch and Canadian student samples [7,8] revealed that a substantial number of drinkers who did not reach the consensus BAC level of 0.11% still reported having a hangover. Other studies also confirmed this observation. For example, the data of van Schrojenstein Lantman et al. [3] identified that 19.4% of N = 1833 students who had a hangover after their past month’s heaviest drinking occasion had an estimated BAC below 0.11%. Data from another survey [9] revealed that 22.5% of N = 989 students had an estimated BAC below 0.11% at their past month’s heaviest drinking occasion that resulted in a hangover. Aggregating the data of these two studies [3,9] revealed that 20.5% of N = 2822 students who reported a hangover had an estimated BAC well below 0.11% the night before (see Figure 1). In each of these studies [3,7,8,9], BAC was (retrospectively) estimated using a modified Widmark formula [10] based on self-reported alcohol consumption, and taking into account sex and body weight.

In non-student samples, alcohol consumption levels are often considerably lower. Nevertheless, these drinkers report having hangovers as well. An illustrative example for this was provided by a recent study conducted among N = 307 adults in Crete, Greece [11]. Among them, N = 176 reported having had a hangover. These individuals were on average 39.0 (10.3) years old (59.7% men) and had consumed a mean (SD) of 3.0 (1.8) alcoholic drinks the previous evening over a drinking period from 17:40 (1.8 h) to 20:13 (1.9 h). Their mean (SD) BAC, estimated via a modified Widmark formula [10], equaled 0.03% (0.03). While the amount of alcohol consumed was low in comparison to student samples, it is still likely that they consumed significantly more alcohol than they usually do at home (i.e., as compared to their usual weekly alcohol intake of 5.9 alcoholic drinks). As a consequence, they reported a mean (SD) being drunk/ intoxicated score of 4.7 (2.6) rated on a scale ranging from 0 (absent) to 10 (extreme) [12,13]. Their mean (SD) overall hangover severity, rated on an 11-point scale ranging from 0 (absent) to 10 (extreme) [14], was 4.6 (2.1). In line with other studies [15,16], both subjective intoxication and estimated BAC correlated significantly with overall hangover severity. The correlations between hangover severity and past evening’s drinking behavior (see Figure 2) revealed that subjective intoxication yielded the strongest correlation with overall hangover severity (Figure 2B), followed by the number of alcoholic drinks consumed (Figure 2A). Although significant, the correlations between hangover severity and estimated BAC (Figure 2C) and drinking duration (Figure 2D) were smaller in magnitude. Figure 2C further shows that participants also reported having hangovers of moderate to high severity at lower BAC levels. In fact, the estimated BAC level of almost all participants (98.3%) fell below 0.11%. 

A stepwise regression analysis revealed that four variables accounted for 58% of the variance in overall hangover severity. When looking at their unique contributions to the variance explained, the strongest predictor was subjective intoxication (48.5%), followed by sleep quality (7.2%), estimated BAC (1.2%), and body mass index (BMI; 1.1%). These findings are in line with two other recent regression analyses [17,18]. Both studies showed that subjective intoxication (perceived drunkenness), and not BAC, was the strongest predictor of hangover severity.

It is important to have an understanding as to why people experience hangovers at low BAC levels. An explanation could be found through a closer examination of the participants’ weekly alcohol consumption. If participants usually consume one or two alcoholic drinks per drinking occasion, and then consume two or three times as much while on holiday, this “higher than usual” drinking level may cause a hangover. This could potentially occur even if the absolute number of alcoholic drinks is still low as compared to some student samples [14]. The increase in the number of consumed alcoholic drinks, as compared to “regular” drinking occasions, and the corresponding increase in subjective intoxication, highly correlates with experiencing a hangover the next day. Importantly, this increase is independent of the absolute BAC levels. In other words, hangovers may occur at any BAC level, and their occurrence is more likely if individuals drink substantially more alcohol than they usually do on occasions that do not result in a hangover.

The impact of an increase in alcohol consumption relative to a regular drinking occasion was also demonstrated in a recent study [19]. This naturalistic study comprised an alcohol test day resulting in a hangover, and an alcohol-free control day. Various demographic data (e.g., age, sex, height, and weight) and data on drinking variables (including number of drinks, drinking duration, and the number of additional drinks they had consumed on the hangover drinking occasion as compared to a regular non-hangover drinking occasion) were collected in students aged 18 to 30 years. BAC was estimated using a modified Widmark equation [10]. Overall hangover severity was rated on an 11-point scale ranging from 0 (absent) to 10 (extreme) [14]. The number of hangover episodes that participants had experienced during the past year was also assessed. Dancing frequency during the drinking occasion was rated as “none”, “sometimes”, “often”, or “almost all the time” and the number of cigarettes smoked, drug use, and total sleep time were also recorded. The Five-Shot questionnaire alcohol screening test was used to analyze general drinking behavior [20]. Personality (i.e., somatization, obsession-compulsion, interpersonal sensitivity, depression, anxiety, hostility, phobic anxiety, paranoid ideation, and psychoticism) was assessed with the Brief Symptom Inventory (BSI) [21]. Risk taking was assessed with the RT18 questionnaire [22]. N = 93 participants were included in this study with a mean (SD) age of 21.0 (2.9) years old, and 41.9% were male. On the alcohol test day, participants consumed 9.2 (4.6) alcoholic drinks over a time period of 6.3 (2.2) hours. They reported consuming 6.5 (4.2) more alcoholic drinks than they would normally consume on a regular non-hangover drinking occasion. Although not assessed in this study, assuming the alcohol was consumed within a similar time frame on both occasions, the increase in alcohol consumption likely corresponded to a significant rise in BAC relative to a regular drinking occasion. Mean (SD) next-day hangover severity was 3.5 (2.5). Although the average estimated BAC was relatively high, i.e., 0.16% (0.09), about one-third of the hungover participants (30.4%) had had an estimated BAC below the 0.11% cutoff level. A stepwise linear regression analysis including all the assessed variables revealed that four variables accounted for 31.7% of the variance in overall hangover severity (See Table 1). The analysis showed that with regard to the unique contribution to variance explained of individual variables, the increase in alcohol consumption relative to a regular drinking occasion was the strongest predictor of hangover severity. 

The findings discussed above do not imply that calculating estimated BAC serves no relevant purpose in future hangover research. Quite the opposite, BAC is a valuable measure that must be implemented in experimental studies. Calculating the estimated BAC enables researchers to administer individual amounts of alcohol that have been adjusted for sex and body weight, in order to achieve comparable BAC levels across study participants who undergo experimentally induced intoxication. BAC assessment also serves an important purpose during the process of recruitment of participants. Whenever a certain dose of alcohol is administered in an experimental study, researchers require a prior estimate of whether a participant will experience a next-day hangover at the designated BAC level. For this purpose, the estimated BAC can be calculated for a regular drinking occasion that usually results in a hangover. Preferably, this estimated BAC should be evaluated for more than one drinking occasion, as a recent analysis of data from an experimental study showed that there is a subset of approximately 20% of study participants for whom there was a substantial intra-individual hangover severity difference between the test days, even when the administered amount of alcohol and achieved BAC where the same [23]. With this prior understanding, the researcher can identify a group of drinkers who are resistant to developing hangovers at the (estimated) BAC level that will be achieved in their experimental study, and exclude these individuals. One could also consider excluding participants who report great intra-individual differences. Alternatively, one could identify individuals who are very sensitive to acute alcohol effects, or already develop hangovers at much lower BAC levels than the designated study BAC. It would be ethically inappropriate to include these individuals and they should be excluded in order to reduce drop-out rate due to anticipated adverse events such as vomiting. Finally, future experiments could also use actual BAC measures to avoid recall bias which may emerge with retrospective recall [24]. Similarly, subjective intoxication ratings could be measured in real time rather than retrospectively. However, research has shown that subjective intoxication, either assessed in real time (while drinking) [25] or the next morning (retrospectively, as in the presented survey data discussed in this paper) both highly correlate with hangover severity. Research on predictors of hangover severity and possible tolerance to hangovers is relatively new [19], and future research should explore the spectrum of additional factors, such as genetics, environment, drinking behaviors, and alcohol metabolism to further understand how variations in BAC (e.g., as a result of drinking more alcohol than usual) influence the presence and severity of alcohol hangovers.

Taken together, the research reviewed here suggests that the level of subjective intoxication and the increase in alcohol consumption relative to a regular drinking occasion are stronger predictors of next-day hangover severity than (estimated) BAC. Furthermore, a substantial number of alcohol drinkers experience hangovers at BAC levels well below 0.11%. Therefore, we argue that the current consensus regarding the BAC 0.11% threshold value as a criterion for having a hangover should be abandoned. 

## Figures and Tables

**Figure 1:**
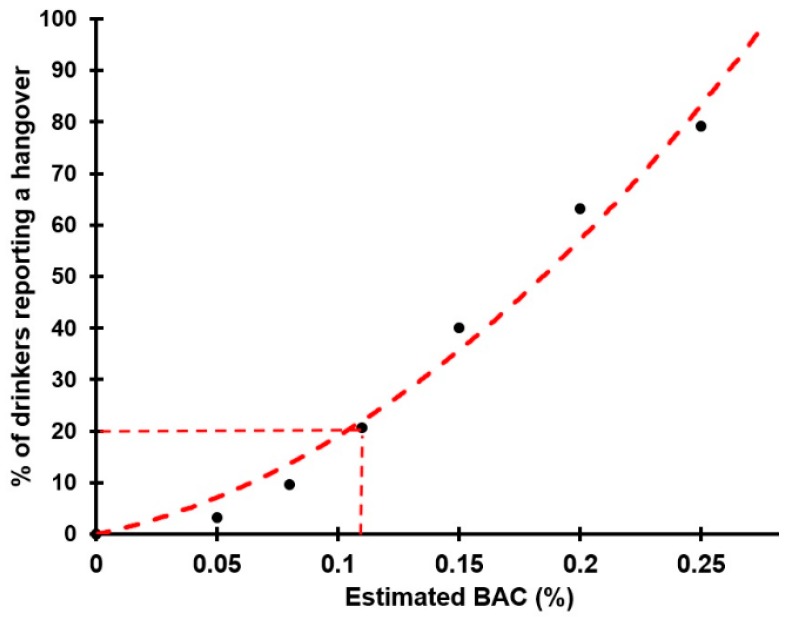
Percentage of students reporting a hangover at different blood alcohol concentrations. Aggregated data from N = 2822 students who reported a hangover after their past month’s heaviest drinking occasion. Data from references [3,9]. Abbreviation: BAC = blood alcohol concentration.

**Figure 2:**
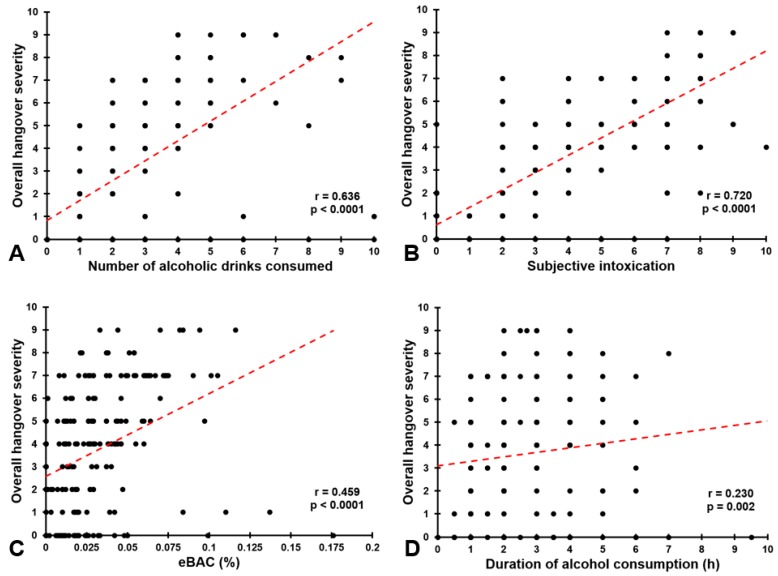
Correlations between overall hangover severity and drinking variables. Depicted are the correlations between overall hangover severity and (**A**) number of alcoholic drinks consumed the previous evening, (**B**) subjective intoxication while drinking, (**C**) estimated blood alcohol concentration (eBAC) on the previous evening, and (**D**) hours of drinking alcohol on the previous evening. Dotted lines represent Spearman’s rho correlations. Data from Reference [11].

**Table 1:** Summary of the regression analysis.

Variables	Model	Contribution
Increase in alcohol consumption relative to a “regular” drinking occasion	17.8%	17.8%
Body mass index (kg/m^2^)	24.0%	6.2%
Dancing frequency on the drinking occasion	28.5%	4.5%
Number of past year’s hangovers	31.7%	3.2%

Variables were included if they significantly (*p* < 0.05) contributed to the model. Significant Spearman’s rho correlations were found between hangover severity and increase in alcohol consumption relative to a regular drinking occasion (r = 0.435, *p* < 0.0001), dancing frequency on the drinking occasion (r = 0.288, *p* = 0.005), and the number of hangovers in the past year (r = 0.529, *p* < 0.0001). The correlation between hangover severity and body mass index did not reach statistical significance (r = −0.144, *p* = 0.168). Data from Reference [18].
